# Role of the Forkhead Transcription Factors Fd4 and Fd5 During *Drosophila* Leg Development

**DOI:** 10.3389/fcell.2021.723927

**Published:** 2021-08-02

**Authors:** Mireya Ruiz-Losada, Cristian Pérez-Reyes, Carlos Estella

**Affiliations:** Centro de Biología Molecular Severo Ochoa (C.S.I.C.-U.A.M.), Universidad Autónoma de Madrid, Madrid, Spain

**Keywords:** Fd4/Fd5, *fd96Ca/fd96Cb*, forkhead transcription factors, leg development, dorso-ventral axis, sex comb, *Drosophila*

## Abstract

Appendage development requires the coordinated function of signaling pathways and transcription factors to pattern the leg along the three main axes: the antero-posterior (AP), proximo-distal (PD), and dorso-ventral (DV). The *Drosophila* leg DV axis is organized by two morphogens, Decapentaplegic (Dpp), and Wingless (Wg), which direct dorsal and ventral cell fates, respectively. However, how these signals regulate the differential expression of its target genes is mostly unknown. In this work, we found that two members of the *Drosophila* forkhead family of transcription factors, Fd4 and Fd5 (also known as *fd96Ca* and *fd96Cb*), are identically expressed in the ventro-lateral domain of the leg imaginal disc in response to Dpp signaling. Here, we analyze the expression regulation and function of these genes during leg development. We have generated specific mutant alleles for each gene and a double *fd4/fd5* mutant chromosome to study their function during development. We highlight the redundant role of the *fd4/fd5* genes during the formation of the sex comb, a male specific structure that appears in the ventro-lateral domain of the prothoracic leg.

## Introduction

Territorial specification depends on the ability of cells to activate a specific developmental program depending on their position within a tissue. The positional information, often provided by extrinsic signaling molecules, is integrated at the *cis*-regulatory modules (CRMs) of genes that encode for transcription factors that instruct cells with a unique developmental fate. Appendage development is a great model to study pattern formation, as it requires the specification of different cell fates along three main axes: the antero-posterior (AP), the dorso-ventral (DV), and the proximo-distal (PD) (Estella et al., 2012; Ruiz-Losada et al., 2018). More than 30 years of studies in *Drosophila* have identified many of the signals and transcription factors that pattern these axes, numerous of them conserved in vertebrates (Shubin et al., 1997; Tabin et al., 1999; Pueyo and Couso, 2005). An important, and yet not fully understood question is how the different signaling pathways regulate the restricted expression of the pattering genes along the three main appendage axes.

**FIGURE 1:**
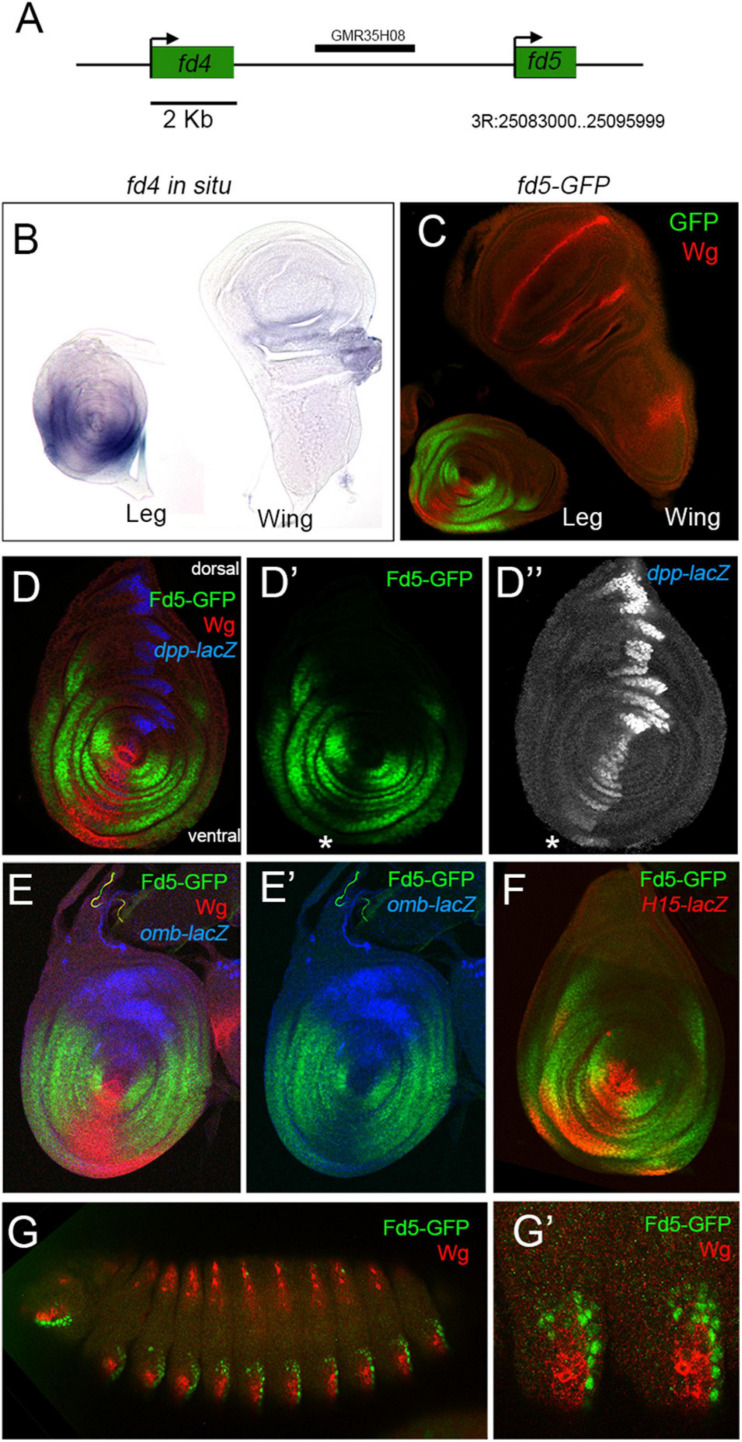
*fd4* and *fd5* expression during development. **(A)** Schematic representation of the *fd4* and *fd5* genomic locus. **(B)** *fd4* expression pattern by *in situ* hybridization. **(C)** *fd5* expression visualized by a *fd5-GFP* tagged (green) line. Wg (red) staining is also shown. **(D)** Expression pattern of *fd5* (green), *dpp-lacZ* (blue), and *wg* (red) in the leg disc. Separate channels for *fd5* **(D′)** and *dpp* **(D″)** are shown. The asterisk indicates the ventral most region of the disc where low Fd5-GFP levels are detected. **(E)** Leg imaginal disc stained for Fd5-GFP (green), *omb-lacZ* (blue), and Wg (red). Separate channels for Fd5-GFP (green) and *omb-lacZ* (blue) are shown in panel **(E′)**. **(F)** Leg imaginal disc stained for Fd5-GFP (green) and *H15-lacZ* (red). **(G)** Stage 14 embryo stained for Fd5-GFP (green) and Wg (red). **(G′)** A close up view of the ventro-lateral domain of two abdominal segments. In all confocal images, dorsal is up and anterior is left.

Appendages in *Drosophila* are derived from specialized epithelial sacs, named imaginal discs, which are specified in the embryo and grow and pattern during larva development (reviewed in Ruiz-Losada et al., 2018). The leg imaginal disc is divided into an anterior and posterior compartment by the expression of the selector gene *engrailed* (*en*) in the posterior compartment. En activates the expression of the short-range morphogene Hedgehog (Hh) in posterior compartment cells. Hh signals to anterior cells where it induces the transcription of two signaling molecules: Decapentaplegic (Dpp) in dorsal anterior cells and Wingless (Wg) in ventral anterior cells (Basler and Struhl, 1994). The dorsal and ventral domains of *dpp* and *wg* expression are maintained by a mutual repression, where Dpp prevents the activation of *wg* in dorsal cells and *vice versa* (Brook and Cohen, 1996; Jiang and Struhl, 1996; Johnston and Schubiger, 1996; Morimura et al., 1996; Penton and Hoffmann, 1996; Theisen et al., 1996). However, low levels of *dpp* expression could still be observed in the ventral domain of the leg disc (Figure 1D). Both, Dpp and Wg are required for the initiation and patterning of the PD and DV axes (Diaz-Benjumea et al., 1994; Brook and Cohen, 1996; Lecuit and Cohen, 1997; Campbell and Tomlinson, 1998). The juxtaposition of cells expressing high levels of Wg and Dpp in the center of the leg disc leads to the formation of the PD axis by activating a regulatory cascade of transcription factors that divide the leg in different domains of gene expression (Estella et al., 2012). In addition to initiating the PD axis formation, Wg and Dpp play an instructive role in the distinction between dorsal and ventral fates (Brook and Cohen, 1996; Jiang and Struhl, 1996; Johnston and Schubiger, 1996; Morimura et al., 1996; Penton and Hoffmann, 1996; Theisen et al., 1996). Dpp specifies dorsal fates and represses ventral ones, while Wg specifies ventral identities and represses dorsal fates. Therefore, hypomorphic mutations of *wg* show strong derepression of Dpp in the ventral domain and formation of ectopic dorsal structures in place of the corresponding ventral ones in the adult leg (Held et al., 1994). Similar phenotypes, but in the opposite direction, were observed in *dpp* mutants (Held et al., 1994). Interestingly, lateral fates are recovered in double hypomorphic mutants for *dpp* and *wg*, suggesting that the lateral fate is the default DV state (Held et al., 1994). The Dpp and Wg signaling molecules depend on the activation of a specific set of transcription factors that promote the DV fate of these cells. The family of T-box transcription factors plays an important role in the specification of DV identities (Brook, 2010). In the ventral domain of the leg, Wg activates while Dpp represses the expression of the redundant genes *H15* and *midline* (*mid*) that act as selector genes for ventral fates, promoting the acquisition of ventral identity. Accordingly, mutants for *H15/mid* lack ventral leg structures or these are transformed to dorsal, whereas when ectopically expressed in the dorsal domain, *H15/mid* induce ventral fates (Svendsen et al., 2009, 2019). In the dorsal domain, the expression of the T-box genes *optomotor-blind* (*omb*) and the *Dorsocross* (*Doc*) *1*, *2*, and *3* are able to repress ventral genes (Maves and Schubiger, 1998; Reim et al., 2003; Svendsen et al., 2015). However, whether *omb* and the *Doc* genes are required to specify dorsal fates is mostly unknown. Gene expression analysis and loss of function studies suggested that the function of the PD and DV patterning genes is generally conserved across arthropods (Maves and Schubiger, 1998; Abzhanov and Kaufman, 2000; Prpic et al., 2001; Inoue et al., 2002; Angelini and Kaufman, 2004; Ober and Jockusch, 2006; Janssen et al., 2008; Grossmann et al., 2009).

In this study we address the role of the sister genes *fd4* and *fd5* (also known as *fd96Ca* and *fd696cb*), members of the forkhead family of transcription factors, during appendage development (Hacker et al., 1992). We found that *fd4/fd5* are expressed exclusively in the ventral imaginal discs and more specifically in the ventro-lateral domain of the leg disc. We identified a minimal CRM that directs the expression of these genes and characterized its regulation in the leg disc. Furthermore, using specific mutations generated for each gene we found that *fd4/fd5* play redundant roles during the formation of the sex comb, a characteristic ventro-lateral structure of the prothoracic male leg. Our results highlight the function of the *fd4/fd5* genes during leg development.

## Results

### *fd4* and *fd5* Expression During Leg Development

To identify genes with a potential role in DV patterning we searched the Flylight database for non-coding DNA elements that have a restricted DV activity pattern in the leg disc (Jory et al., 2012). We identified a DNA fragment that activates the reporter gene in the ventro-lateral domain of the leg disc (named GMR35H08 at Flylight database). This fragment is located between the *fd4* and *fd5* genes, two members of the forkhead family of transcription factors (Hacker et al., 1992; Lee and Frasch, 2004; Figure 1A). The Fd4 and Fd5 proteins share a 49% of aa sequence identity, suggesting they could play similar functions. In order to investigate the expression pattern of these genes we used an *in situ* hybridization probe for *fd4* and GFP-tagged versions for *fd4* and *fd5*. Both genes are identically expressed in the three leg imaginal discs, the antenna and the genital imaginal discs and show no expression in the wing or haltere discs (Figures 1B,C and Supplementary Figure 1; Heingard et al., 2019). When compared to the dorsal and ventral determinants, *dpp* and *wg*, *fd4* and *fd5* expression is restricted to the ventro-lateral domain of the leg disc with faint expression in the ventral most region that coincides with the highest levels of Wg and low Dpp (Figure 1D and Supplementary Figure 1; Heingard et al., 2019). Comparison of *fd5* expression with that of the Dpp and Wg targets, *omb* and *H15*, confirmed that *fd5* expression is complementary to *omb* and extends more laterally than *H15* (Estella and Mann, 2008; Brook, 2010; Ruiz-Losada et al., 2018; Figures 1E,F). As previously reported, these genes are also expressed in the embryo (Archbold et al., 2014), and at least for *fd5*, its expression is restricted to ectodermal segmental ventral stripes surrounding *wg* expression (Figure 1G).

### Fd4 and Fd5 Act Redundantly in the Formation of the Sex Comb Structure

**FIGURE 2:**
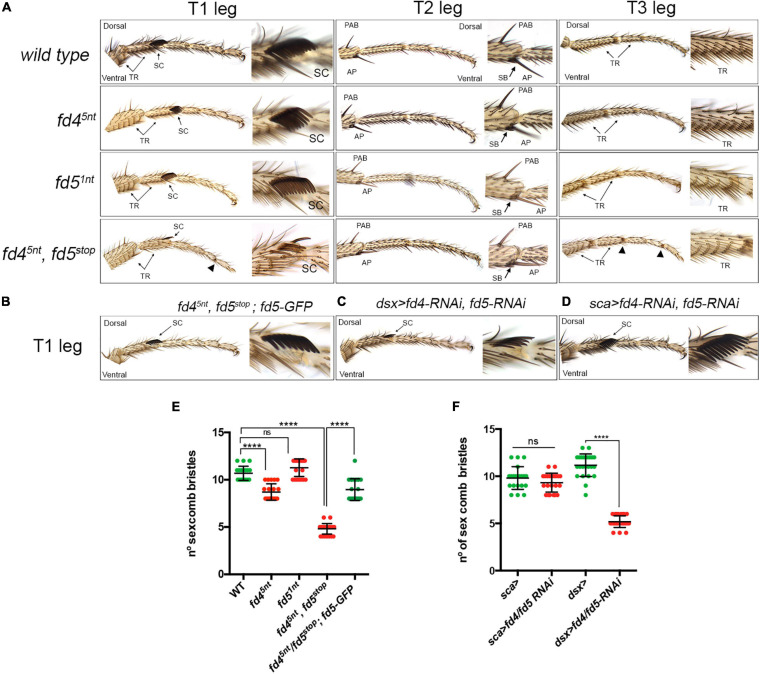
Phenotypic analysis of *fd4* and *fd5* mutants. **(A)** Male adult leg phenotypes of a wild type, *fd4^5*nt*^*, *fd5^1*nt*^* and the double *fd4^5*nt*^*, *fd5*^*stop*^ mutants. Representative examples of the three legs (T1–T3) are shown. Close up views of the sex comb in T1, the distal tibia in T2, and the first tarsus in T3 are shown. TR, transverse row; SC, sex comb; PAB, pre-apical bristle; AP, apical bristle and SB, spur bristles. Arrowheads indicate defective joints. Characteristic ventro-lateral structures are the transverse rows (TRs), the sex combs (SCs), and the apical bristles (ABs). **(B–D)** Male first thoracic legs of the genotypes indicated. Note that the sex comb phenotype of the double *fd4^5*nt*^*, *fd5*^*stop*^ mutant is largely rescued by one copy of the *fd5-GFP* transgene **(B)**. Knockdown of Fd4 and Fd5 levels with the *dsx-Gal4* **(C)**, but not with the *sca-Gal4* **(D)**, reduced the number of sex comb bristles. **(E,F)** Quantification of sex comb bristles in the genotypes indicated and presented in panels **(A,B)**. *n* > 19 sex combs per genotype were counted. Error bars indicate standard deviation (SD). Statistically significant differences based on Student’s *t* test are indicated: ****P < 0.0001 and not significant (ns).

Next, we investigated the role of *fd4* and *fd5* during leg development. To this end, we generated specific mutant alleles for each gene and a double *fd4/fd5* mutant using CRISPR/Cas9 (Supplementary Figure 2) (see details in Materials and Methods). The *fd4^5*nt*^* mutant allele has a five nucleotide (nt) deletion at the beginning of the coding region that changes the open reading frame. *fd4^5*nt*^* homozygous mutant flies are viable and have normal patterned legs with the exception of a slight reduction in the number of sex comb bristles (∼10.6 in the control vs. ∼8.7 in *fd4^5*nt*^* mutants) (Figures 2A,E). The sex comb is a male specific structure present on the prothoracic leg (T1 leg) that develops from modified bristles of the most distal transverse row (TRs) on the first tarsal segment that rotate approximately 90° (Tokunaga, 1962; Tanaka et al., 2009; Kopp, 2011). The *fd5^1*nt*^* mutant has a reading frame shift due to a single nt deletion at the start of the gene that completely changes the amino acid sequence (Supplementary Figure 2). No defects were observed in the legs of *fd5^1*nt*^* mutant animals (Figures 2A,E). As both genes have identical expression patterns, and likely similar functions, we generated a double *fd4*, *fd5* mutant chromosome by mutating the *fd5* gene over the *fd4^5*nt*^* allele (Supplementary Figure 2). The new *fd5*^*stop*^ mutant has a nine nt sequence change and a three nt deletion that generate a premature stop codon at the beginning of the coding region (Supplementary Figure 2). *fd4^5*nt*^*, *fd5*^*stop*^ homozygous mutant animals reach adulthood, though they get caught in the food where they die soon afterwards.

A detailed leg phenotypic analysis of the different DV landmarks present in the three legs from *fd4^5*nt*^*, *fd5*^*stop*^ double mutant animals revealed defects in the formation of the sex comb and some necrotic tissue in few joints (Hannah-Alava, 1958). We also found that the pattern of transverse row bristles is slightly altered in these mutants (Figure 2A and Supplementary Figure 3). The number of sex comb bristles is strongly reduced in the double mutant *fd4^5*nt*^*, *fd5*^*stop*^ when compared to each single mutant or the control (∼10.6 in the control vs. ∼4.8 in *fd4^5*nt*^*, *fd5*^*stop*^ mutants), suggesting a redundant role of these genes in the formation of this male specific structure (Figures 2A,E). The orientation of the remaining sex comb teeth is longitudinal as in the control, suggesting that the 90° rotation of precursor distal transverse row bristles have occurred properly in the mutants. In females, we detected approximately the same number of ta1 transverse rows in the control and in the double *fd4^5*nt*^*, *fd5*^*stop*^ mutant (Supplementary Figure 3). Importantly, the number of sex combs bristles of *fd4^5*nt*^*, *fd5*^*stop*^ mutant animals was almost completely rescued when a wild type copy of the *fd5* gene (BAC-*fd5*-GFP) was provided in the mutant background (∼4.8 in *fd4^5*nt*^*, *fd5*^*stop*^ mutants vs. ∼9 in the *fd4^5*nt*^*, *fd5*^*stop*^; *fd5-GFP* rescue), confirming that these phenotypes are specific of the *fd4*, *fd5* mutations (Figures 2B,E).

In addition, we used specific RNAi lines for each gene that efficiently reduced Fd4 and Fd5 protein levels (Supplementary Figure 4). When these RNAi lines were expressed in the distal domain of the leg with the *Dll-Gal4* driver (*Dll*>) we obtained identical phenotypes that with the mutants (Supplementary Figure 4). These results corroborate the redundant roles of the *fd4* and *fd5* genes in the formation of the sex comb.

Next, we investigated whether the requirement of the *fd4/fd5* genes is restricted to the bristles precursors that will form the sex comb teeth or to the epidermal cells of the leg imaginal disc. To this end, we used the *scabrous* (*sca*)-*Gal4* line to knock down simultaneously Fd4 and Fd5 levels in nascent bristle cells (Shroff et al., 2007). In addition, both RNAi lines were simultaneously expressed with the *doublesex* (*dsx*)-*Gal4* that is expressed both in the epidermis and sensory organ precursors (SOPs) of the sex comb (Robinett et al., 2010). While no defects were observed in *sca*>*fd4-RNAi*, *fd5-RNAi* animals, a strong reduction in the number of sex comb bristles was found in *dsx*>*fd4-RNAi*, *fd5-RNAi* flies (∼11 in *dsx*> control animals vs. ∼5.2 in *dsx*>*fd4/fd5-RNAi* mutant animals) (Figures 2C,D,F). These results suggest that Fd4 and Fd5 function is not restricted to late sex comb SOPs but instead required in leg epithelium cells including those that will be re-specified as SOPs.

**FIGURE 3:**
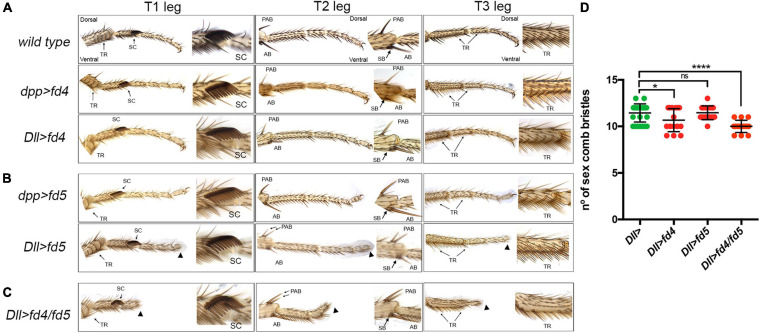
Phenotypic analysis of *fd4* and *fd5* gain of function. **(A,B)** Male adult legs phenotypes of wild type control animals and of flies misexpressing *fd4* **(A)** or the *fd5* **(B)** in the distal (*Dll-Gal4*) or dorsal (*dpp-Gal4*) domain of the legs. Representative examples of the three legs (T1–T3) are presented. Close up views of the sex comb in T1, the distal tibia in T2, and the first tarsus in T3 are shown. Arrowheads indicate distal truncations. Arrows indicate the presence of duplicated PABs. **(C)** Male adult legs of flies misexpressing *fd4* and *fd5* under the *Dll-Gal4* driver. Note the severe truncation of the distal leg (arrowheads) and the presence of two PABs. **(D)** Quantification of sex comb bristles in the genotypes indicated and presented in panels **(A–C)**. *n* = 15 sex combs per genotype were counted. Error bars indicate standard deviation (SD). Statistically significant differences based on Student’s *t* test are indicated: *P < 0.05, ****P < 0.0001, and not significant (ns).

In order to study the function of the *fd4/fd5* genes in gain of function experiments we have generated specific UAS lines for each gene and ectopically expressed them in the dorsal region (*dpp-gal4*) or in the entire distal domain (*Dll-Gal4*) of the leg imaginal disc (Figure 3). Importantly, the ectopic expression of *fd4* or *fd5* did not increase the number of sex comb bristles in any of these conditions (Figures 3A,B,D). Nevertheless, we observed the appearance of an extra dorsal pre-apical bristle in 50% of T2 legs and a distal truncation that deletes the claw in the *Dll*>*fd5* genotype (Figure 3B). When both genes were expressed together with the *Dll*-*Gal4* line, a more severe distal truncation phenotype was detected, however the number on sex comb bristles remained close to the control (∼11.4 in *Dll*> vs. ∼10 in *Dll*>*fd4/fd5* animals) (Figures 3C,D).

In summary, all these results suggest that *fd4/fd5* act redundantly in the formation of the sex comb, however these genes are not sufficient to generate ectopic sex comb teeth when ectopically expressed.

### Analysis of *fd4/fd5* Role in the Sex Comb Regulatory Network

**FIGURE 4:**
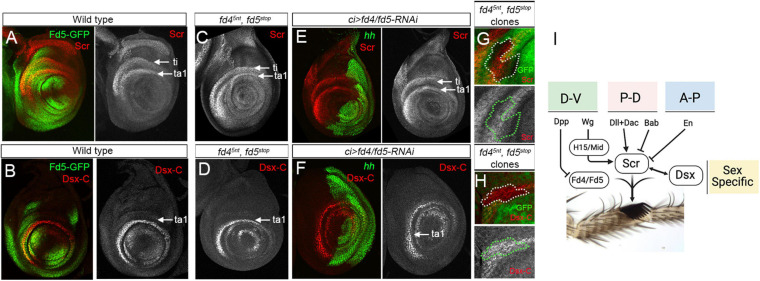
Fd4/Fd5 are not requiered for *Scr* and *dsx* expression. **(A,B)** Expression of *fd5*-*GFP* (green) and *Scr* (red, **A**) or *dsx-C* (red, **B**) in a male third instar prothoracic leg imaginal disc. Single channels for Scr and Dsx-C stainings are shown. White arrows point to the tibia and first tarsal segment. **(C,D)** Scr **(C)** and Dsx-C **(D)** antibody stainings in male prothoracic leg imaginal discs of *fd4^5*nt*^*, *fd5*^*stop*^ mutant animals. **(E,F)** Male prothoracic leg imaginal discs stained for Hh (green) and Scr (red, **E**) and Dsx-C (red, **F**). The knockdown of Fd4/Fd5 levels in the anterior compartment by the expression of the *fd4* and *fd5 RNAi* lines under the *ci-Gal4* driver does not affect Scr or Dsx-C. Single channels for Scr and Dsx-C staining are shown. **(G,H)** *fd4^5*nt*^*, *fd5*^*stop*^ mitotic mutant clones visualized by the absence of GFP (green) and by a dotted white and green line. Scr **(G)** and Dsx-C **(H)** levels (red) are not affected in *fd4^5*nt*^*, *fd5*^*stop*^ mutant cells. Single channels for Scr and Dsx-C staining are shown. **(I)** The sex comb integrate multiple regulatory inputs form the three axes (D-V, P-D, and A-P) and sex-specific cues to develop in a precise location in the first tarsal segment of the male prothoracic leg. A simplified version of the gene regulatory network that directs the formation of the sex comb is depicted. In all confocal images, dorsal is up and anterior is left.

The formation of the sex comb is directed by a gene regulatory network that precisely localizes this structure in the anterior ventro-lateral domain of the first tarsal segment of prothoracic male legs (Kopp, 2011). PD patterning genes such as *Dll*, *dachsound* (*dac*), and *bric à brac* (*bab*), in combination with *H15/mid*, *wg*, and *en* regulate the prominent expression of the Hox gene *Sex comb reduced* (*Scr*) in the tibia (ti) and first tarsal segment (ta1) of the prothoracic leg (Tokunaga, 1961; Struhl, 1982; Couderc et al., 2002; Svendsen et al., 2009; Eksi et al., 2018; Figures 4A,I). Scr, together with PD transcription factors, regulate *doublesex* (*dsx*) expression in two anterior distal crescents (ta1 and ta2). Dsx is a sex-specific transcription factor that exists in two isoforms. The male isoform promotes male-specific structures while the female isoform dictates the corresponding female ones (Burtis and Baker, 1989). Once activated, Dsx modulates the sexual dimorphic male-specific expression of *scr* in ta1 segment (Kopp, 2011; Tanaka et al., 2011; Figures 4B,I).

Initially, we analyzed the expression of *wg* and its target genes *H15* and *mid* in *fd4/fd5* loss of function conditions. No changes were observed in the expression of any of these genes (Supplementary Figure 5). Next, as both Scr and Dsx direct the morphogenesis of the sex comb structure, we decided to study the functional relationship between these genes and *fd4/fd5*. To monitor *dsx* expression we used an antibody against the common domain shared by the male and female Dsx protein isoforms (Sanders and Arbeitman, 2008). First, we compared the expression of *fd5-GFP* with Scr and Dsx and confirmed that both genes overlap with Fd5 in the ventro-lateral domain of the presumptive ta1 in male prothoracic imaginal disc, the region that will form the transverse row bristles and the sex comb (Figures 4A,B). Second, we analyzed *Scr* and *dsx* expression in leg discs from *fd4^5*nt*^*, *fd5*^*stop*^ double mutant animals. No visible changes were observed in the expression of these genes in mutants as compared to controls, or when we knockdown Fd4/Fd5 levels in the anterior compartment using the RNAi lines (Figures 4C–F). To confirm that the *fd4/fd5* genes are not modulating Scr or Dsx levels, we generated mosaic mitotic *fd4^5*nt*^*, *fd5*^*stop*^ mutant clones and monitored Scr and Dsx levels in mutant and wild type adjacent cells of the same leg imaginal disc (Figures 4G,H). We did not detect any change on Scr and Dsx levels in *fd4^5*nt*^*, *fd5*^*stop*^ mutant clones. As *fd4* and *fd5* expression is neither sexually dimorphic nor restricted to the prothoracic legs, it is very unlikely that these genes are downstream of Scr and Dsx regulation.

These results indicate that the *fd4/fd5* genes may work in parallel with Scr and Dsx in the regulatory network that controls the formation of the sex comb (Figure 4I).

### Identification of the Leg Disc *fd4/fd5* Minimal *Cis*-Regulatory Module

**FIGURE 5:**
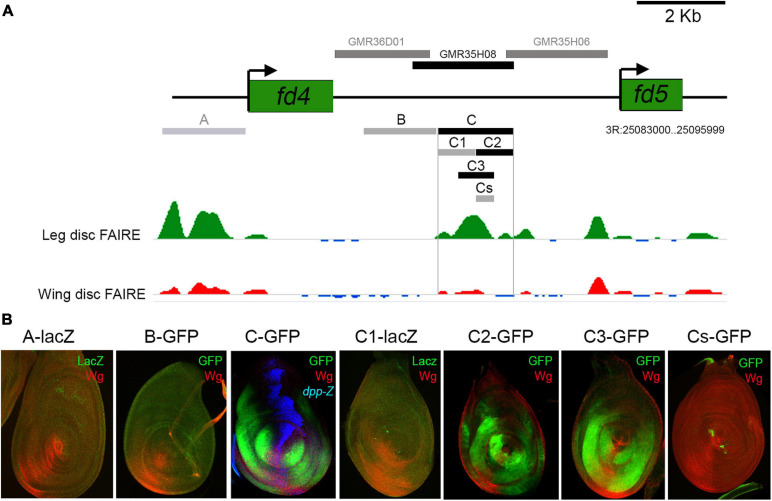
Identification of the *fd4/fd5* CRM. **(A)** Schematic representation of the *fd4* and *fd5* genomic locus in which open chromatin regions, identified by FAIRE seq for leg and wing imaginal discs are indicated by green and red peaks, respectively. Data obtained from McKay and Lieb (2013). In the upper part of the panel, horizontal bars represent the DNA elements for which Gal4 drivers were generated by the Janelia Farm consortium (gray bars). Only the GMR35H08 line (black bar) reproduced *fd4/fd5* expression in the leg disc. Below the genomic locus are drawn the different DNA elements cloned in this work into a reporter *GFP* or *lacZ* construct. Only the C fragment and the C2 and C3 subfragments faithfully reproduced the expression of *fd4/fd5* in the leg imaginal disc. Note that the A and C fragments were selected because of the different chromatin accessibility profiles between the leg and wing imaginal discs. Gray bars indicate no activity and black bars indicate activity in leg imaginal discs. **(B)** Leg imaginal disc activity of the different fragments cloned in this work and shown in panel **(A)**. All elements were cloned in a *GFP* or *lacZ* reporter vector and inserted in the same attP site. GFP (green), Wg (red), and *dpp-lacz* (blue).

To identify the CRMs that direct *fd4/fd5* expression in the leg imaginal disc, we searched for differentially open chromatin regions between the leg and the wing disc in the *fd4/fd5* genomic locus (McKay and Lieb, 2013). Two regions, A and C, show a clear open chromatin state in the leg as opposed to the wing disc (Figure 5A). Both regions and an additional sequence (B fragment) that has been previously shown to reproduce *fd4/fd5* expression in the embryo were cloned in a GFP or lacZ reporter vector (Figures 5A,B; Archbold et al., 2014). Of these three elements, only fragment C, which is contained within the GMR35H08 element, is active in the leg disc in a similar pattern as *fd4/fd5* expression (Figure 5B). However, unlike the *fd4/fd5* genes, this element is also active in the wing disc (Supplementary Figure 6). We further subdivided the C element in two non-overlapping halves (C1 and C2) and a fragment that encompass the peak of open chromatin in the leg (C3) (Figure 5A). As both C2 and C3 elements reproduce *fd4/fd5* expression, we tested the activity of the 200 bp overlapping region (Cs) in the leg disc. No reporter activity was observed for the Cs fragment, suggesting that the C3 element contains the minimal information to drive *fd4/fd5* expression in the leg disc (Figure 5B).

### Regulation of *fd4/fd5* Expression in the Leg Imaginal Disc

**FIGURE 6:**
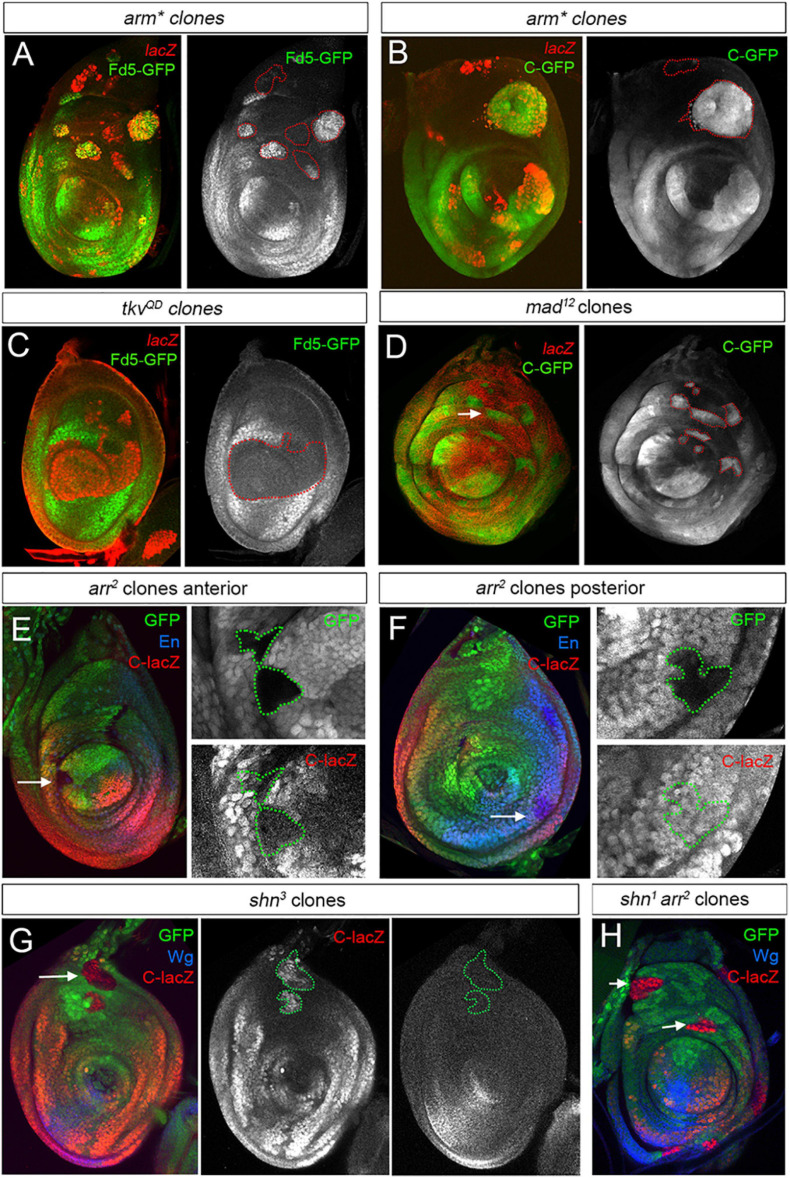
Wg and Dpp contribution to *fd4/fd5* regulation in the leg disc. **(A,B)** Clones expressing arm* marked by lacZ (red) activates *fd5-GFP* (green, **A**) or C-GFP (green, **B**) expression in dorso-lateral regions of the disc. Note that dorsal-most clones failed to activate *fd5* or C activity. Single channels for Fd5-GFP and C-GFP are shown separately and arm* clones are outlined with red dots. **(C)** Clones expressing *tkv*^*QD*^ marked by lacZ (red) repress *fd5-GFP* (green) expression. Single channel for Fd5-GFP is shown and *tkv*^*QD*^ clones are outlined with red dots. **(D)** *mad*^12^ clones marked by absence of lacZ (red), show C-GFP (green) derepression in the dorso-lateral domain of the leg (white arrow). Single channel for C-GFP is shown and clones are outlined with red dots. **(E,F)** *arr*^2^ clones marked by absence of GFP stained for C-lacZ (red) and En (blue) to mark the posterior compartment. In panel **(E)**, an anterior *arr*^2^ mutant clones in the ventro-lateral domain of the leg show downregulation of C-lacZ (arrow). In panel **(F)**, the same clones located in the posterior compartment have no effect on C-lacZ activity (arrow). Single channels for GFP and C-lacZ are shown and clones are outlined with green dots. **(G)** *shn*^3^ mutant clones marked by the absence of GFP (green) derepress C-lacZ activity (red) in the dorsal domain of the leg disc (arrow). Note that in these clones, *wg* expression (blue) is not activated. Single channels for C-lacZ and Wg are shown and clones are outlined with green dots. **(H)** *shn*^1^, *arr*^2^ double mutant clones marked by the absence of GFP (green) derepress C-lacZ activity (red) in the dorsal domain of the leg disc (arrows). Wg staining is in blue. All clones were generated 48–72 h AEL.

As the expression of the *fd4/fd5* genes is restricted to the ventro-lateral domain of the leg disc, we investigated the role of the Dpp and Wg pathways in their regulation. We used the *fd5-GFP* line or the *fd4/fd5* C-CRM as readouts for *fd4/fd5* expression depending on the experimental setup. To test the idea that the *fd4/fd5* genes integrate the Wg and Dpp inputs, we generated clones expressing a constitutive activated form of the β-catenin homolog Armadillo (Arm^∗^) to activate the Wg pathway, or an activated form of the Dpp receptor Thickveins (Tkv^*QD*^), respectively (Figures 6A–C). Most of dorsally located *arm*^∗^ expressing clones show a cell autonomous upregulation of *fd5-GFP* and *C-GFP* CRM expression (Figures 6A,B). However, *arm*^∗^ clones close to the *dpp* domain failed to induce the expression of these reporters (Figures 6A,B). In contrast, activation of the Dpp pathway by *tkv*^*QD*^ expressing clones strongly repressed *fd5-GFP* expression (Figure 6C). These results suggest that the Wg pathway activates, while the Dpp pathway represses *fd4/fd5* expression. To test for the requirement of these pathways in the regulation of the *fd4/fd5* genes, we generated mitotic loss of function clones for the transcriptional effectors of the Dpp pathway, Mad and for the Wg co-receptor Arrow (Arr). *C-GFP* was strongly upregulated in all dorsally located *mad*^12^ clones, while ventral *arr*^2^ mutant clones exhibit different behaviors of C-CRM activity depending on their localization (Figures 6D–F). For example, ventral anterior clones close to the AP compartment border downregulate C-*lacZ* activity while ventral posterior clones have no effect on it (Figures 6E,F, respectively). The regulation of *fd4/fd5* expression by the Dpp and Wg pathways is not mediated by their target genes *omb* and *H15/mid*, as mutant clones for these genes have no effect on *fd5-GFP* expression or C activity (Supplementary Figure 6).

Decapentaplegic and Wg transcriptionally repress each other in the leg disc, and therefore the downregulation of one pathway allows the activation by Hh of the other in anterior cells (Brook and Cohen, 1996; Jiang and Struhl, 1996; Johnston and Schubiger, 1996; Morimura et al., 1996; Penton and Hoffmann, 1996; Theisen et al., 1996). Our *arr*^2^ mutant clone analysis points to an indirect regulation of *fd4/fd5* expression through the derepression of the Dpp pathway instead of a direct requirement of the Wg pathway for its expression. To further test this possibility, we first generated mutant clones for Schnurri (Shn), a transcriptional repressor downstream of Dpp activity. Shn is a zinc finger protein that together with Mad/Med forms a complex that regulates Dpp target genes such as *brinker* (*brk*) (Arora et al., 1995; Grieder et al., 1995; Marty et al., 2000). Dorsally located *shn*^3^ mutant clones activated the expression of the *C-lacZ* reporter cell autonomously and, importantly, they do so without derepression of *wg* (Figure 6G). Next, we induced *shn*^1^ mutant clones that are also mutant for *arr*, and therefore cannot transduce the Wg pathway. Consistently with our previous results, these *shn*^1^ *arr*^2^ mutant clones still derepressed C-*lacZ* activity in dorsal leg regions (Figure 6H).

Taken together, these results demonstrate that *fd4/fd5* expression is repressed dorsally by the Dpp effectors Shn and Mad and is activated independently of Wg.

**FIGURE 7:**
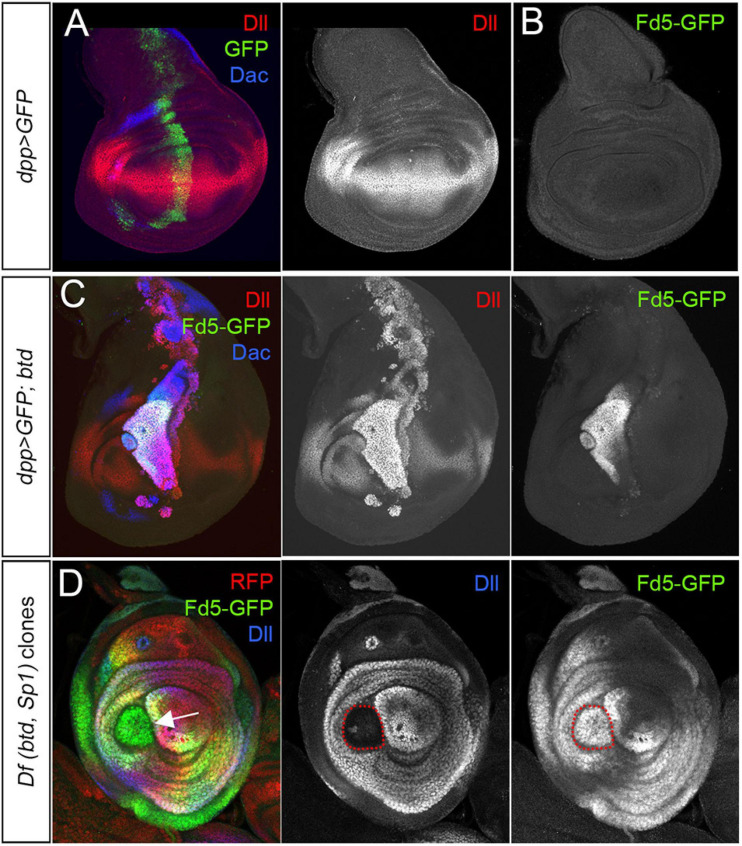
*fd5* regulation by *btd* and *Sp1*. **(A)** Control wing imaginal disc expressing GFP (green) under the *dpp-Gal4* driver and stained for Dll (red) and Dac (blue). Separate channel for Dll is shown. **(B)** Wing imaginal disc from a *fd5-GFP* line. Note the absence of *fd5* expression (green) in the wing disc. **(C)** The expression of *btd* under the control of the *dpp-Gal4* line ectopically activates *fd5-GFP* (green) expression in the wing disc. Dll (red) and Dac (blue). Separate channels for Dll and Fd5-GFP are shown. **(D)** *Df(btd*, *Sp1)* mutant clones generated 48–72 h before dissection marked by the absence of RFP (red and arrow) and stained for Fd5-GFP (green) and Dll (blue). Single channels for Dll and Fd5-GFP are shown. Clones are outlined with red dots.

*fd4/fd5* are expressed in ventral (legs and antenna) but not dorsal (wing and haltere) imaginal discs, suggesting that their expression could be regulated by a positive input from a ventral selector gene. The sister genes, *buttonhead* (*btd*) and *Sp1* could fulfill this role: in one hand, forced expression of *btd* or *Sp1* in the wing disc induces ectopic leg development and in the other, removing *btd* and *Sp1* completely abolish leg formation (Estella et al., 2003; Estella and Mann, 2010). To test this idea, we analyzed *fd5-GFP* expression in wing discs where *btd* is ectopically induced (Figures 7A–C). Misexpression of *btd* in the wing imaginal disc with the *dpp-Gal4* line activates *fd5-GFP* in the wing pouch but not in the notum (Figures 7A–C). In contrast, clones for a deficiency that deletes the *btd* and *Sp1* genes (*Df(btd,Sp1)*) in the leg disc has no effect on *fd5-GFP* expression, even when these clones lose the ability to activate *Dll* (Figure 7D). These results suggest that although *btd* is sufficient to ectopically induce *fd5-GFP* expression in the wing disc, *btd* and *Sp1* are not necessary for endogenous *fd5* expression in the leg disc.

## Discussion

In this work we studied the expression and function of the forkhead family members *fd4* and *fd5* during leg development in *Drosophila*. We found that these genes play redundant roles during sex comb formation.

Subdivision of the DV territories is regulated in a different manner between wing and leg appendages. For example, in the wing imaginal disc the expression of the selector gene *apterous* (*ap*) is activated in response to the epidermal growth factor receptor (EGFR) pathway and it is required for the specification of dorsal cell fates (Diaz-Benjumea and Cohen, 1993; Blair et al., 1994; Wang et al., 2000; Bieli et al., 2015). In the leg imaginal disc, DV subdivision is controlled by Dpp and Wg signaling pathways that direct dorsal and ventral fates, respectively (Brook and Cohen, 1996; Jiang and Struhl, 1996; Johnston and Schubiger, 1996; Morimura et al., 1996; Penton and Hoffmann, 1996; Theisen et al., 1996). As Dpp and Wg form gradients, an interesting problem is to understand how cells in the leg disc, which are exposed to different levels of these morphogens are able to integrate this information and assume dorsal, ventral, and lateral fates. This is of special interest as the leg is a circular appendage with no clear morphological DV distinction and no lineage restriction as opposed to the wing. Moreover, in contrast to the PD axis, we have very limited information of the downstream Wg and Dpp targets that controls DV patterning in the leg.

In a search for genes with DV expression patterns in the leg, we identified the forkhead family members *fd4* and *fd5* to be restricted to the ventro-lateral domain of the ventral imaginal discs. In contrast to other known DV leg patterning genes, the *fd4/fd5* expression is extended more laterally than the Wg target genes *H15/mid*. These genes are not activated in the dorsal domain of the leg, where high levels of Dpp are present. In addition, we identified a minimal CRM that faithfully recapitulates *fd4/fd5* expression in the leg imaginal disc. Detailed analysis of the C element regulation reveals that this CRM is activated by an unknown factor and repressed by the Dpp pathway, more specifically by the transcriptional repressor Shn. Interestingly, we found that the C element is similarly expressed as *brk*, another Shn target gene (Marty et al., 2000; Estella and Mann, 2008). However, Brk is not required for C element activity in the leg as the *C-GFP* CRM is normally expressed in *brk* mutant clones (Supplementary Figure 6). No consensus binding site for the Mad/Med/Shn complex was found in the C-CRM sequence, suggesting that either this complex is binding a non-consensus site or that *fd4/fd5* regulation by Shn is indirect (Pyrowolakis et al., 2004). Importantly, we found that the Wg pathway is indirectly required for C element activity through the repression of Dpp expression.

Unlike the endogenous expression of the *fd4/fd5* genes, the C element is not restricted to the ventral imaginal discs (legs and antenna), as activity of this CRM is also observed in the wing disc. This result suggests that sequences outside the C and GMR35H08 elements restrict *fd4/fd5* expression to the ventral imaginal discs. We studied the potential role of the ventral selector genes Sp1 and Btd as regulators of *fd4/fd5* ventral specific expression. However, *btd/Sp1* loss of function clones still display *fd5* expression in the leg. It is possible that wing disc specific genes are required for repressing *fd4/fd5* expression in dorsal imaginal discs and restricting its activity to ventral ones.

According to their identical expression and sequence homology, we found that these genes play redundant roles during leg development. Using newly generated alleles for each gene and a double *fd4/fd5* mutant, we describe a redundant role for these genes in sex comb formation. In contrast to their wide lateral expression in all three legs, we only found defects on the development of this specific male structure of the first thoracic leg. In males, the distal most transverse row of the first tarsal segment is transformed into a sex comb. We found that in *fd4/fd5* double mutants the number of sex comb bristles is strongly reduced but not eliminated, suggesting that these genes contribute to sex comb development but are not completely required. As the sex comb is formed from cells with ventral and lateral fates (Held et al., 1994), the remaining sex comb bristles observed in the *fd4/fd5* mutants would only be formed from cells with ventral identity.

The sex comb gene regulatory network integrates information from the three axes (AP, DV, and PD) and sex- and segment-specific cues by the *dsx* and *Scr* genes, respectively (Kopp, 2011). No defects on the expression of *H15/mid*, *Scr*, or *dsx* were observed when *fd4/fd5* levels were knock down, suggesting that the *fd4/fd5* act in parallel to these genes in the regulatory network that controls sex comb formation. The sex comb is a great model to study how the precise combination of positional and sex specific patterning cues promote the formation of morphological structures. Understanding the genetics of sex comb development could help understand the origin and diversification of this recently evolved structure.

A recent study has analyzed the expression and function of the forkhead transcription factor FoxB in the common house spider *Parasteatoda tepidariorum* (Heingard et al., 2019). This gene is the ortholog of *fd4/fd5* and the only family member in the spider. Similar to *Drosophila fd4/fd5*, *pt-FoxB* expression is restricted to the ventral domain of developing appendages and it is required for DV patterning. In *pt-Foxb-RNAi* animals the expression of the ventral determinants *wg* and *H15* is almost lost while the corresponding ventral expansion for the dorsal determinants, *dpp* and *omb*, is described. It is remarkable the different mutant phenotypes observed in the spider and the fly after the knockdown of these genes. One possibility that could explain these differences is that the double *fd4^5*nt*^*, *fd5*^*stop*^ mutant chromosome generated in our study is not a true null for *fd5* but a strong hypomorph due to stop codon readthrough mechanisms (Palma and Lejeune, 2021). Either way, the leg phenotypes described here using the double *fd4^5*nt*^*, *fd5*^*stop*^ mutant and the RNAi lines are almost identical. Also, in the *Parasteatoda* study the authors used RNAi techniques to downregulate the function of the *Foxb* gene. Another possibility is that the function and requirement of the FoxB forkhead transcription factors have been modified during the evolution of arthropods. Thanks to the development of new technologies such as CRISPR/Cas9 the function of the FoxB forkhead transcription factors could be easily investigated in other arthropods to study how the DV gene regulatory network has been modified in related species.

## Materials and Methods

### *Drosophila* Strains

*fd4-GFP* (*P{fd96Ca-GFP.FPTB}attP40*), *fd5-GFP* (*P{fd96Cb-GFP.FPTB}attP40*), *dpp^10368^-lacZ (p10368)*, *omb-lacZ*, *H15-lacZ*, *mid-lacZ*, *dpp-Gal4*, *UAS-GFP*, *Dll^212^-Gal4*, *dsx-Gal4*, *sca-Gal4*, *UAS-fd4-RNAi*, *UAS-fd5-RNAi* and *hh-dsred*, *Ci-Gal4* are described in FlyBase.

Loss-of-function clonal analysis the following genotypes were used:

*yw122*; *FRT82B fd4^5*nt*^*, *fd5^*stop*^/yw122*; *FRT82B-ubi-GFP*.

*yw122*; *FRT42D arr^2^/w122*; *FRT42D ubi-GFP*; *C-lacZ*

*yw122*; *Mad^1–2^ FRT40A/arm-lacZ FRT40A*; *C-GFP*

*yw122*; *FRT42D shn^3^*; *C-lacZ/yw122*; *FRT42D ubi-GFP*.

*yw122*; *FRT42D arr^2^ shn^1/^yw122*; *FRT42D ubi-GFP*; *C-lacZ*

*Df(btd,Sp1) FRT19A/yw122 ubi-RFP FRT19A*, *fd5-GFP*

*omb^282^ FRT19A/yw122 ubi-RFP FRT19A*, *fd5-GFP*

*yw122*; *H15^*X*4^ mid^1*a*5^ FRT40A/ubi-GFP FRT40A*; *C-lacZ*

*brk^*M*68^ FRT19A/yw122 ubi-RFP FRT19A*, *C-GFP*

Clones were induced by heat shocking 48–72 h AEL (after egg laying) larvae for 1 h at 37°C.

Gain-of-function clonal analysis:

*yw122*; *act*>*STOP*>*Gal4*, *UAS-lacZ; fd5-GFP/UAS-tkv^*QD*^*

*yw122*; *act*>*STOP*>*Gal4*, *UAS-lacZ; fd5-GFP/UAS-arm^∗^*

*yw122*; *act*>*STOP*>*Gal4*, *UAS-lacZ; C-GFP/UAS-arm^∗^*

Clones were induced by heat shocking 48–72 h AEL larvae for 10 min at 37°C.

At least three clones were scored for each experiment.

All fly lines listed above are described in FlyBase.

### Generation of the *fd4^5*nt*^*, *fd5^1*nt*^* and *fd4^5*nt*^*, *fd5*^stop^ Mutant Flies

The *fd4^5*nt*^*, *fd5^1*nt*^* and *fd4^5*nt*^*, *fd5*^*stop*^ mutant flies were generated by CRISPR-Cas9 technique. Briefly, *fd4* and *fd5* gRNAs were cloned in the pBFv-U6.2 and injected in *y^1^v^1^ P{y[* + *t7.7]* = *nos-phiC31\int.NLS}X;P{y[* + *t7.7]* = *CaryP}attP40* flies. These flies were then crossed with flies expressing Cas9 in their germinal line cells. Candidate mutants were confirmed by sequencing. gRNAs sequences were selected as the produced mutations (insertions and deletions) at the beginning of the respective gene. The following sequences were used as primers to clone the gRNAs in the pBFv-U6.2 vector following FlyCas9 protocols^1^ :

*Sense fd4* 5′-CTTCGTAGGATTCTCGCGAGGGCC-3′

*Asense fd4* 5′-AAACGGCCCTCGCGAGAATCCTAC-3′

*Sense fd5* 5′-CTTCGCTGAGCGGCAGCAATCTTTG-3′

*Asense fd5* 5′-AAACCAAAGATTGCTGCCGCTCAGC-3

To generate the *fd4^5*nt*^*, *fd5*^*stop*^ double mutant we introduced the *fd5* gRNA into *fd4^5*nt*^* flies. The candidate mutant flies were sequenced to verify the new mutations produced in the *fd5* gene.

### Generation of UAS-*fd4* and UAS-*fd5* Lines

Wild type *fd4* and *fd5* DNA was amplified from genomic DNA and cloned in the pUAST attB vector using the following primers (restriction sites are underlined and restriction enzyme used is noted in brackets):

For UAS-*fd4*:

5′-CAGTGAATTCGTAATAATGCCCCGGCCCTC GCGAGAATCC-3′ (*Eco*RI)

5′-CAGTCTCGAGCCCTGCTTGTTGCCACTTATCTAT ATCGTACGC-3′(*Xho*I)

For UAS-*fd5*:

5′ CAGTAGATCTCTTCGCAATGCCACGCCC ATTGAAAATGAG 3′ (*Bgl*II)

5′ CAGTGCGGCCGCTCAAAAGACGGGCAACGGGCCG 3′ (*Not*I)

Both UAS constructs were inserted into the same attP site (86Fb).

### Generation of *GFP* and *lacZ* Reporter Transgenic Lines

To generate the A, B, C, C1, C2, C3, and Cs reporter constructs, DNA from the *fd4/fd5* locus was amplified by PCR from genomic DNA. For the GFP reporter lines, the DNA fragments were first cloned into the pEntry/D-TOPO vector and then swapped into the *attB-pHPdesteGFP* vector (Boy et al., 2010), using the LR Clonase Enzyme Mix (Thermo Fisher Scientific). For the *lacZ* reporter lines, DNA fragments were amplified with primers containing restriction enzyme sites as overhangs, and subsequently cloned into plasmid *attB-hs43-nuc-lacZ* (Giorgianni and Mann, 2011).

We cloned all the sequences in the *GFP* reporter vector except for the C1 and A fragments that were cloned in the *lacZ* vector and the C-CRM that was cloned in both vectors.

To carry out these experiments, we used the following primers (restriction sites are underlined and restriction enzyme used is noted in brackets)

A fragment in *attB-hs43-nuc-lacZ* vector:

5′-CAGTGCGGCCGCCCGTGGCCATATTCATATGTCCAC-3′ (*Not*I)

5′-CAGTGCGGCCGCAGGATTCTCGCGAGGGCCGGGG-3′(*Not*I)

B fragment in *attB-pHPdesteGFP* vector:

5′CACCGCGCACCAGGCCACGCCCACCCCCG-3′

5′CAGTAGATCTGGAGCCGCAGGGGCCAGATA-3′

C fragment in *attB-hs43-nuc-lacZ* vector:

5′-CAGTGCGGCCGCGTATCTGGCCCCTGCGGCTCC-3′(*Not*I)

5′CAGTAGGCCTGCCCAGAGGCGGATTCGGATTCGG-3′(*Stu*I)

C fragment in *attB-pHPdesteGFP* vector:

5′-CACCGTATCTGGCCCCTGCGGCTCC-3′

5′-GCCCAGAGGCGGATTCGGATTCGG-3′

C2 fragment in *attB-pHPdesteGFP* vector:

5′-CACCCAGATCTCAGTTTCTCGGTTCG-3′

5′-GCCCAGAGGCGGATTCGGATTCGG-3′

C3 fragment in *attB-pHPdesteGFP* vector:

5′-CACCAGATCTCGGCTCGGGTGTTGATGC-3′

5′-CAGTGCGGCCGCGCCGCTAGTTGCCGGCAC-3′

Cs fragment in *attB-pHPdesteGFP* vector:

5′-CACCCAGATCTCAGTTTCTCGG-3′

5′-CAGTGCCGCTAGTTGCCGGCAC-3′

The C1-lacZ fragment was cloned from the C sequence included in the *attB-hs43-nuc-lacZ* vector, using the restriction site of *Not*I enzyme that had been included at the beginning of C sequence, and *Bgl*II that digests in the middle of the C fragment. The primers to clone the C2 fragment were designed to clone from *Bgl*II.

All these constructs were inserted into the same attP site (86Fb).

### Immunostaining

Embryos and larval leg imaginal discs were fixed and stained following standard procedures described in Requena et al. (2017). We used the following primary antibodies: rabbit anti-βGal (1/1000; Promega), mouse anti-βGal (1/1000; MP Biomedicals), guinea pig anti-Dll (1/2000) (Estella et al., 2008), rabbit anti-GFP (1/1000; Thermo Fisher Scientific), mouse anti-Dac (1/50; DSHB #mAbdac1-1), mouse anti-Wg (1/50; DSHB #4D4), rat-anti-Dsx-C (1/200) (Sanders and Arbeitman, 2008), mouse-anti-Scr (1/50; DSHB #6H4.1) and mouse anti-En (1/50; DSHB #4D9).

### *In situ* Hybridization

The *fd4* RNA probe used in this experiment was generated by PCR from genomic DNA using primers with the recognition sequences of the RNA polymerase T7 and T3:

Forward T7:

5′-TAATACGACTCACTATAGGGGGGACT GACCAATCTGCCCGCGC-3′

Reverse: T3:

5′-ATTAACCCTCACTAAAGGGACGGGGCTCCG ATATTGCTGCGCC-3′

The transcription to generate antisense probes was done using the RNA polymerase T3 in the presence of DIG (DIG RNA labeling mix, Roche) at 15°C for 2 h, and the probes were precipitated and suspended in H_2_O with DEPC (Sigma).

Imaginal discs were dissected in PBS 1× and fixed in 4% formaldehyde for 30 min at room temperature. Then, we washed in PBS-0.1% Tween (PBT) three times, and refixed for 20 min at room temperature in 4% formaldehyde, 0.1% Tween. The samples were washed again in PBT.

After three washes in PBT, discs were incubated overnight at –20°C in hybridization solution (SH; 50% formamide, SSC 5×, 100 μg/ml salmon sperm DNA, 50 μg/ml heparin, and 0.1% Tween).

The next day, we washed the disc in SH for 10 min at room temperature. The discs were prehybridized for 2 h at 55°C in SH and hybridized with digoxigenin (DIG)-labeled RNA probes at 55°C. The probes were previously denatured at 80°C for 10 min. All the solutions used before hybridization were treated with DEPC (Sigma). After hybridization, discs were washed in SH and PBT and incubated for 2 h at room temperature in a 1:4000 dilution of anti-DIG antibody (Roche). After incubation, the discs were washed in PBT and the detection of probes was performed with 4-Nitro blue tetrazolium chloride (NBT) and 5-Bromo-4-chloro-3-indolyl-phosphate (BCIP) solution (Roche). The discs were mounted in 70% glycerol.

## Data Availability Statement

The raw data supporting the conclusions of this article will be made available by the authors, without undue reservation.

## Author Contributions

MR-L, CP-R, and CE contributed to the conception and design of the study, performed the experiments, and analyzed the data. CE wrote the manuscript. All authors contributed to manuscript revision, read, and approved the submitted version.

## Conflict of Interest

The authors declare that the research was conducted in the absence of any commercial or financial relationships that could be construed as a potential conflict of interest.

## Publisher’s Note

All claims expressed in this article are solely those of the authors and do not necessarily represent those of their affiliated organizations, or those of the publisher, the editors and the reviewers. Any product that may be evaluated in this article, or claim that may be made by its manufacturer, is not guaranteed or endorsed by the publisher.

## Footnotes

1
https://shigen.nig.ac.jp/fly/nigfly/cas9/

## Supplementary Material

Supplementary Figure 1 *fd4* and *fd5* expression in ventral imaginal discs. Third instar eye-antenna, genital and leg imaginal discs stained for Fd4-GFP (green) or Fd5-GFP (green), *dpp-lacZ* (blue), and Wg (red).

Supplementary Figure 2 Generation of *fd4*, *fd5* and *fd4*, *fd5* double mutant alleles. Schematic representation of the different mutants generated for this study. gRNA target sites are in red. Chromatograms, as determined by Sanger sequencing, for the evaluation of the different mutants are presented. All sequences were obtained from homozygous flies at the exception of the *fd5*^1nt^ mutant. Insertions are indicated in blue. Asterisks indicate the first stop codon generated. Gray boxes represent alterations in the protein sequence due to stop codons and open reading frame changes. The corresponding amino acid sequences are shown below.

Supplementary Figure 3 *fd4* and *fd5* mutant’s leg phenotypes. Adult male and female prothoracic legs of wild type, *fd4*^5nt^, *fd5*^1nt^ and the double *fd4*^5nt^, *fd5*^*stop*^ mutants. The tibia (tib) and the first tarsal (ta1) segment are shown. Sex comb (SC) are indicated, and numbers mark the transverse rows in the ta1.

Supplementary Figure 4 Validation of *fd4* and *fd5* RNAi lines. **(A–D)** Third instar leg imaginal discs stained for GFP (green) and *hh-dsRED* (red) of the following genotypes: **(A)** *ci-Gal4*, *hh-dsRED*; *Fd4-GFP*, **(B)** *ci-Gal4*, *hh-dsRED*; *UAS-fd4-RNAi*; *Fd4-GFP*, **(C)** *ci-Gal4*, *hh-dsRED*; *Fd5-GFP*, and **(D)** *ci-Gal4*, *hh-dsRED*; *UAS-fd5-RNAi*; *Fd5-GFP*. Separate channels for GFP and Hh are shown. Note the downregulation of Fd4 and Fd5 levels in the anterior compartment in panels **(B,D)**. The compartment boundary is marked by white dots. **(E)** Male prothoracic adult legs of the indicated genotypes. Close up views of the sex comb are shown. **(F)** Quantification of sex comb bristles in the genotypes indicated and presented in panel **(E)**. *n* > 15 sex combs per genotype were counted. Error bars indicate standard deviation (SD). Statistically significant differences based on Student’s *t* test are indicated: ^****^*P* < 0.0001 and not significant (ns).

Supplementary Figure 5 Fd4/Fd5 are not required for *H15* and *mid* expression. **(A)** Leg imaginal disc stained for Fd5-GFP (green), Wg (blue), and *mid-lacZ* (red). **(B)** Expression of the *fd4* and *fd5* RNAi lines under the control of the *Dll-Gal4* line to knockdown Fd4/Fd5 levels. Leg imaginal disc is stained for *mid-lacZ* (red) and Wg (blue). The domain of *Dll-Gal4* is marked by green dots. **(C)** The knockdown of Fd4/Fd5 levels in the anterior compartment by the expression of the *fd4* and *fd5* RNAi lines under the *ci-Gal4* has no effect on *H15-lacZ* expression (green) or Wg protein (blue). *hh* expression is in red and the compartment boundary is marked by red dots. In panels **(A–C)**, separate channels for *mid-lacZ*, *H15-lacZ*, and Wg are shown.

Supplementary Figure 6 *brk*, *omb*, and *mid/H15* mutant clones have no effect on *fd4/fd5* expression. **(A)** C-GFP activity is observed in the wing imaginal disc in a complementary pattern to *dpp* expression and similar to *brk* (not shown). **(B)** *brk*^*M68*^ mutant clones in the leg disc marked by the absence of RFP (red) and stained for C-GFP (green). Separate channels for RFP and C-GFP are shown and the *brk*^*M68*^ mutant clones are outlined by white dots. **(C)** Leg imaginal disc with a dorsal *omb*^282^ mutant clone marked by the absence of RFP (red, arrow) and stained for Fd5-GFP (green) and Wg (blue). Separate channel for Fd5-GFP is shown and the clone is outlined by a red dotted line. **(D)** *H15^*X4*^ mid^1a5^* mutant clones marked by the absence of GFP (green) in a third instar leg imaginal disc stained for C-lacZ (red) and Wg (blue). An arrow marks a ventro-lateral anterior compartment clone and a close up view is shown. All clones were generated 48–72 h before dissection.
