# Supplementary figures and images for: Role of the Forkhead Transcription Factors Fd4 and Fd5 During Drosophila Leg Development
Source: Front Cell Dev Biol. 2021 Aug 2;9:723927. doi: 10.3389/fcell.2021.723927 (PMC8365472; doi:10.3389/fcell.2021.723927)

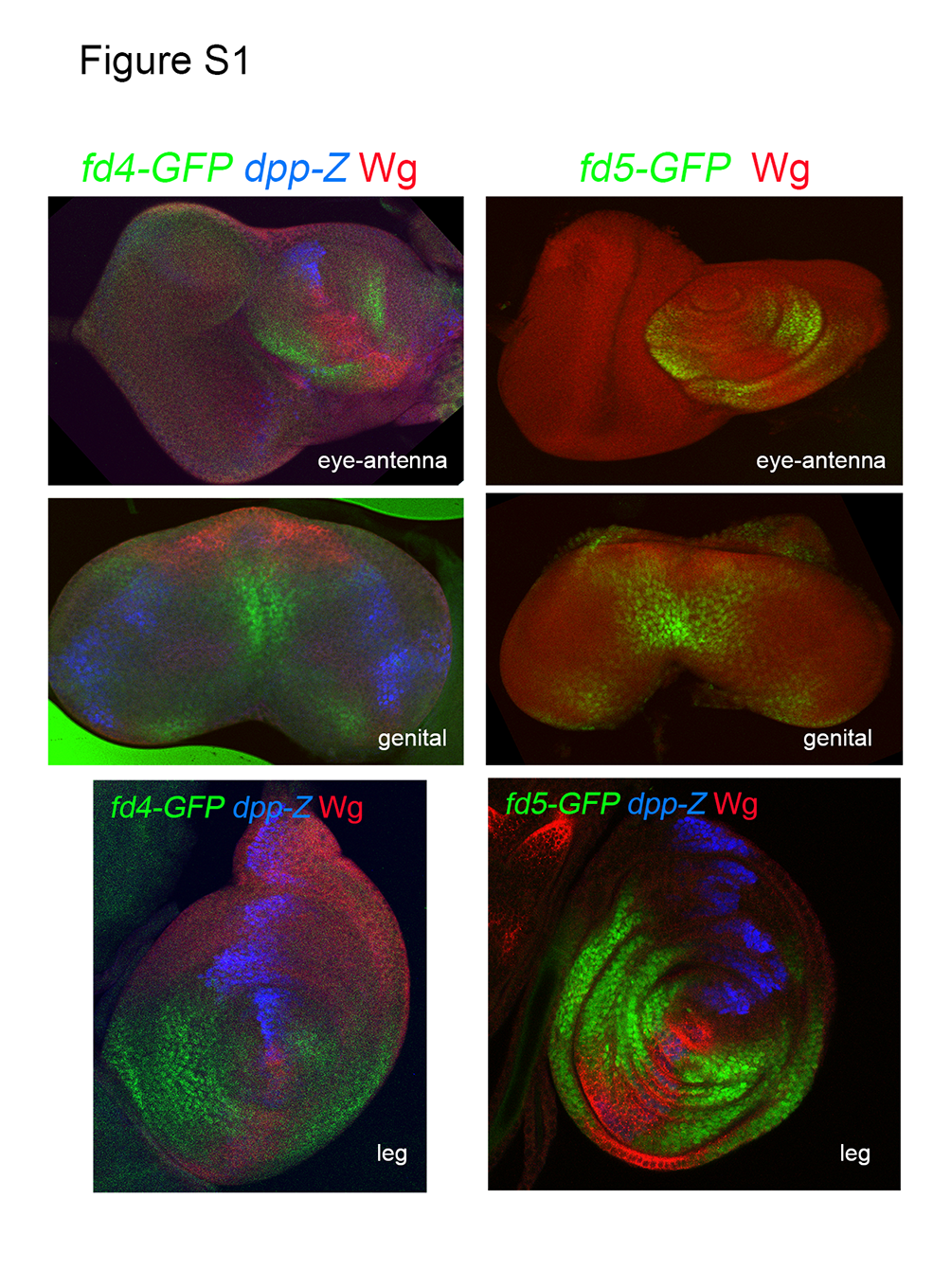

Supplement: Supplementary Figure 1 — fd4 and fd5 expression in ventral imaginal discs. Third instar eye-antenna, genital and leg imaginal discs stained for Fd4-GFP (green) or Fd5-GFP (green), dpp-lacZ (blue), and Wg (red). [file Image_1.TIF]

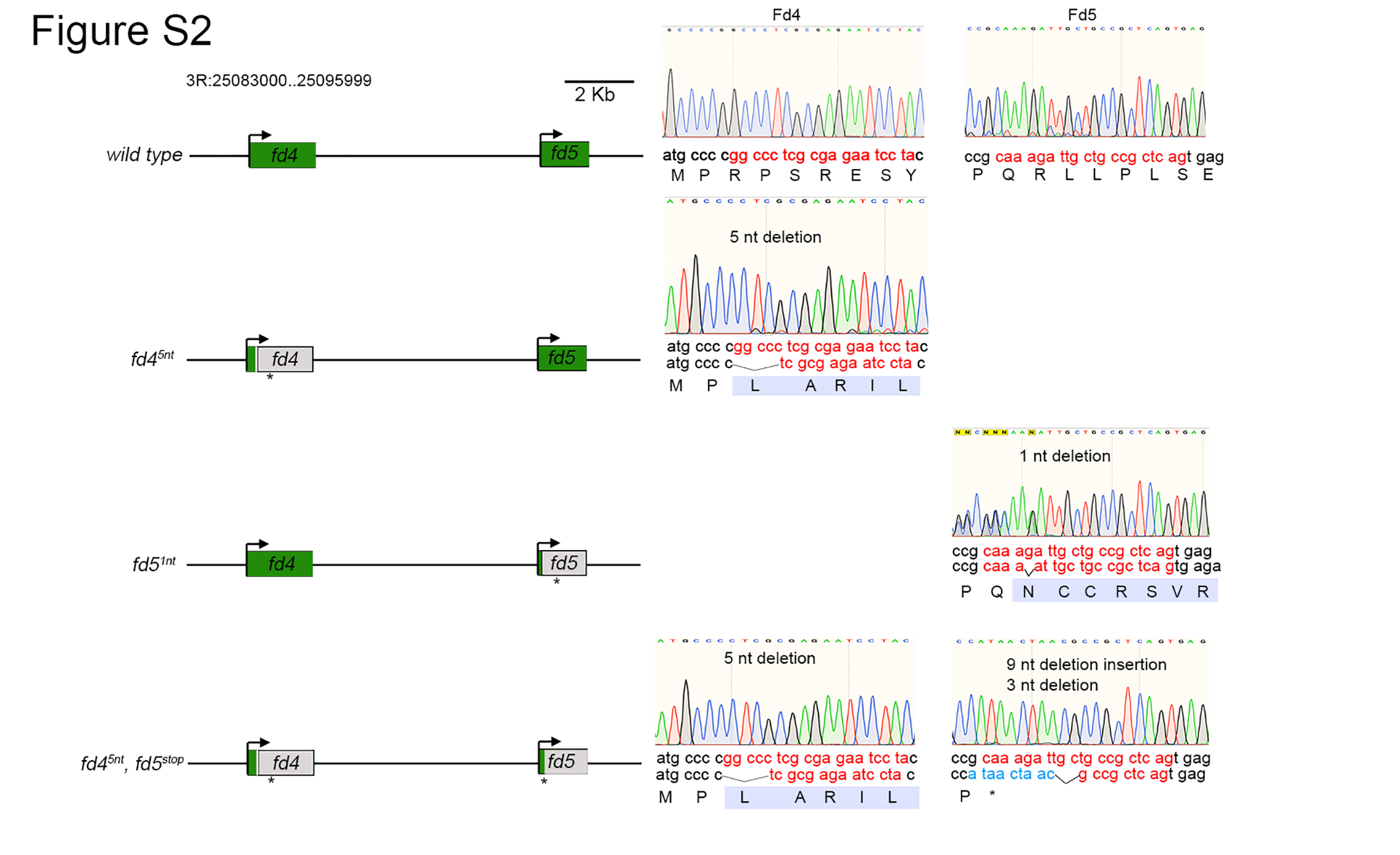

Supplement: Supplementary Figure 2 — Generation of fd4, fd5 and fd4, fd5 double mutant alleles. Schematic representation of the different mutants generated for this study. gRNA target sites are in red. Chromatograms, as determined by Sanger sequencing, for the evaluation of the different mutants are presented. All sequences were obtained from homozygous flies at the exception of the fd51nt mutant. Insertions are indicated in blue. Asterisks indicate the first stop codon generated. Gray boxes represent alterations in the protein sequence due to stop codons and open reading frame changes. The corresponding amino acid sequences are shown below. [file Image_2.TIF]

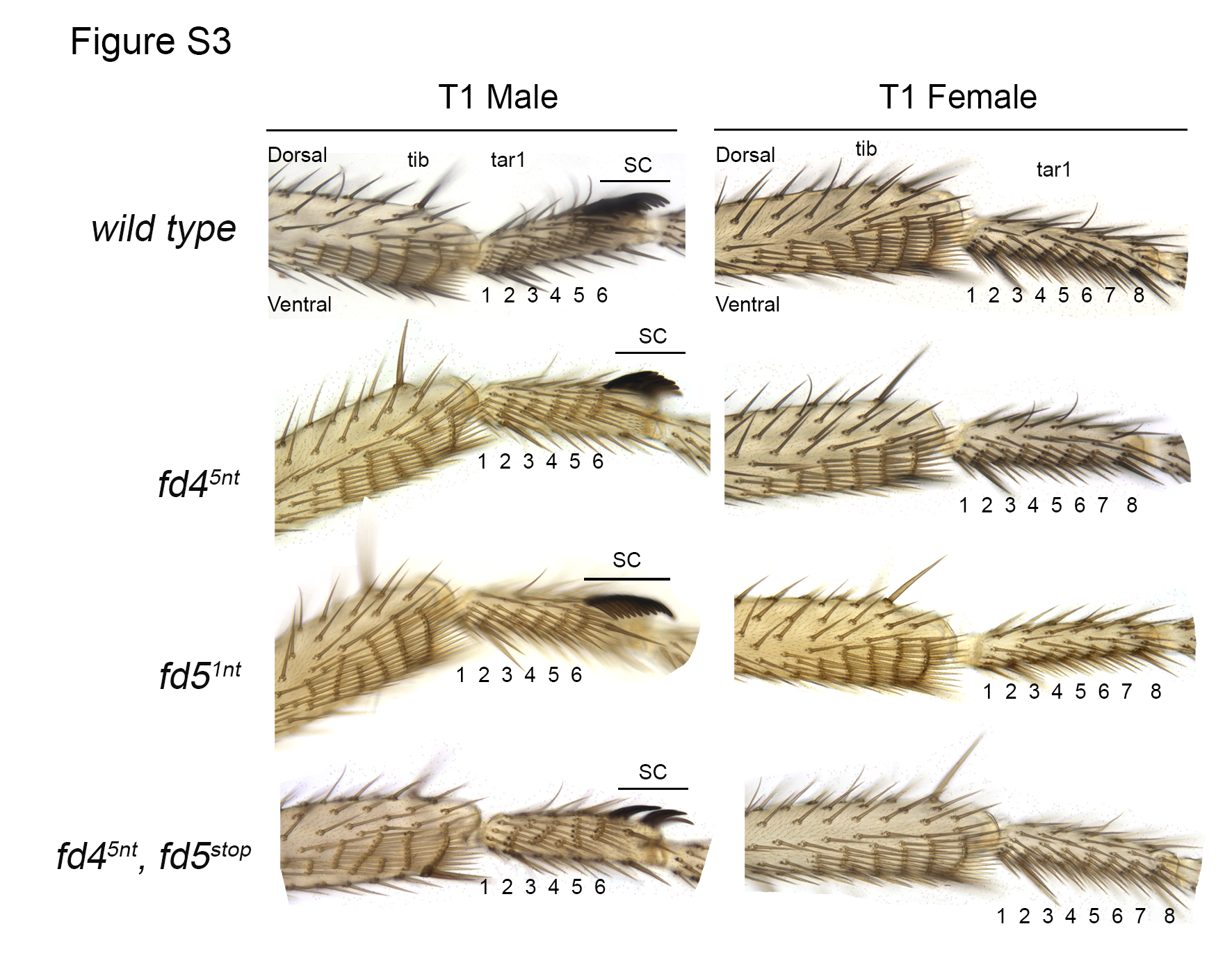

Supplement: Supplementary Figure 3 — fd4 and fd5 mutant’s leg phenotypes. Adult male and female prothoracic legs of wild type, fd45nt, fd51nt and the double fd45nt, fd5stop mutants. The tibia (tib) and the first tarsal (ta1) segment are shown. Sex comb (SC) are indicated, and numbers mark the transverse rows in the ta1. [file Image_3.TIF]

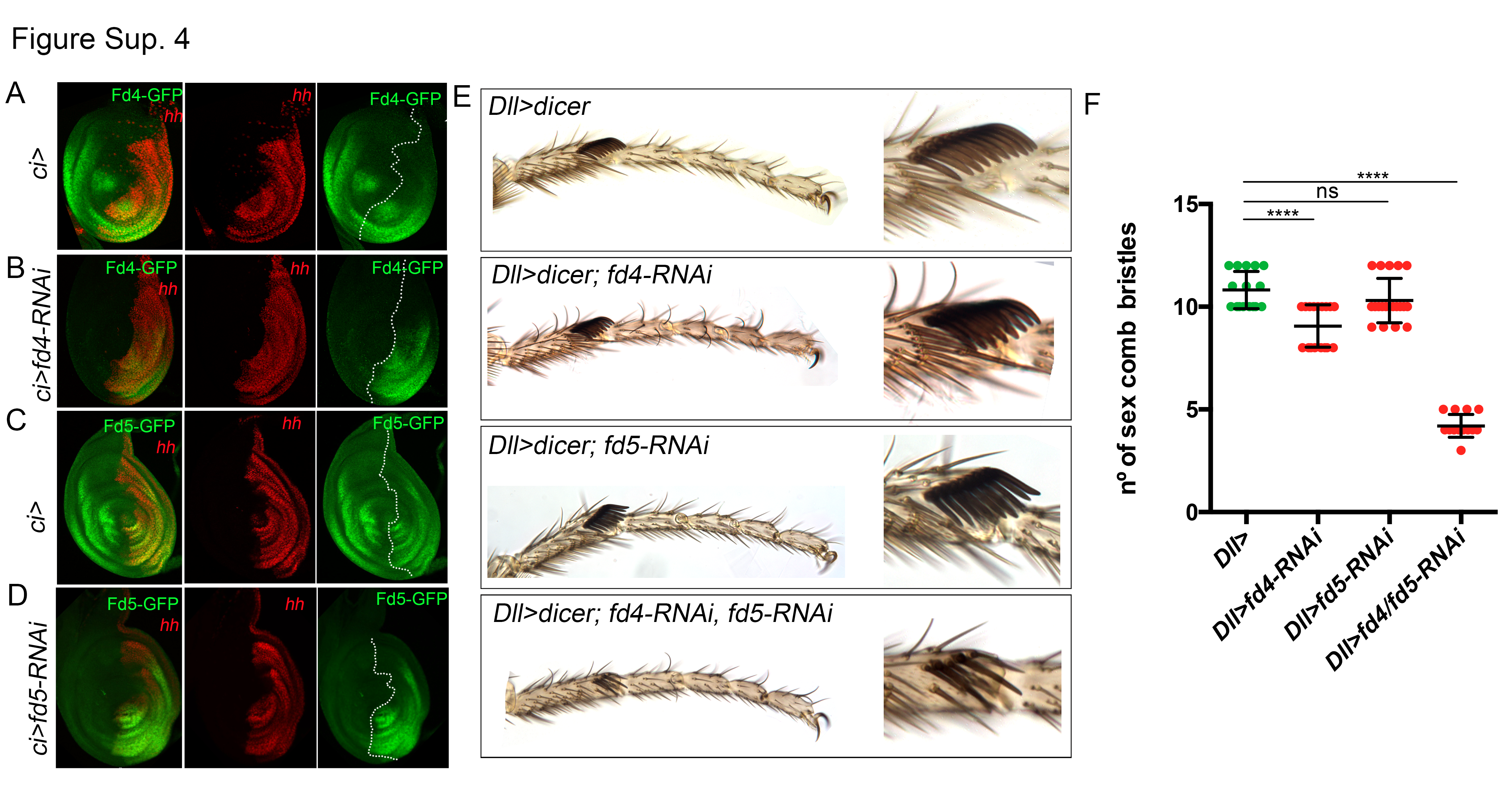

Supplement: Supplementary Figure 4 — Validation of fd4 and fd5 RNAi lines. (A–D) Third instar leg imaginal discs stained for GFP (green) and hh-dsRED (red) of the following genotypes: (A) ci-Gal4, hh-dsRED; Fd4-GFP, (B) ci-Gal4, hh-dsRED; UAS-fd4-RNAi; Fd4-GFP, (C) ci-Gal4, hh-dsRED; Fd5-GFP, and (D) ci-Gal4, hh-dsRED; UAS-fd5-RNAi; Fd5-GFP. Separate channels for GFP and Hh are shown. Note the downregulation of Fd4 and Fd5 levels in the anterior compartment in panels (B,D). The compartment boundary is marked by white dots. (E) Male prothoracic adult legs of the indicated genotypes. Close up views of the sex comb are shown. (F) Quantification of sex comb bristles in the genotypes indicated and presented in panel (E). n > 15 sex combs per genotype were counted. Error bars indicate standard deviation (SD). Statistically significant differences based on Student’s t test are indicated: ****P < 0.0001 and not significant (ns). [file Image_4.TIF]

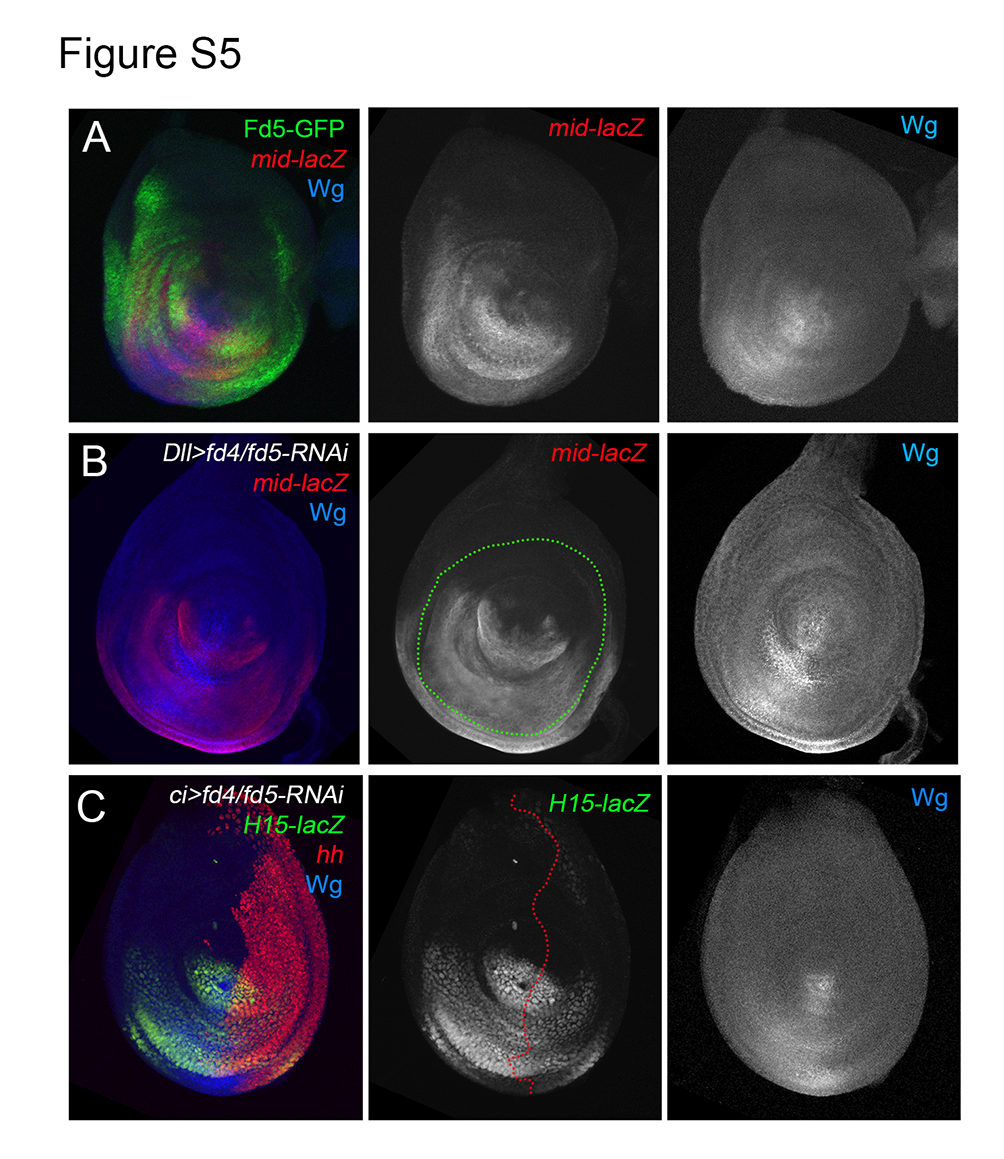

Supplement: Supplementary Figure 5 — Fd4/Fd5 are not required for H15 and mid expression. (A) Leg imaginal disc stained for Fd5-GFP (green), Wg (blue), and mid-lacZ (red). (B) Expression of the fd4 and fd5 RNAi lines under the control of the Dll-Gal4 line to knockdown Fd4/Fd5 levels. Leg imaginal disc is stained for mid-lacZ (red) and Wg (blue). The domain of Dll-Gal4 is marked by green dots. (C) The knockdown of Fd4/Fd5 levels in the anterior compartment by the expression of the fd4 and fd5 RNAi lines under the ci-Gal4 has no effect on H15-lacZ expression (green) or Wg protein (blue). hh expression is in red and the compartment boundary is marked by red dots. In panels (A–C), separate channels for mid-lacZ, H15-lacZ, and Wg are shown. [file Image_5.TIF]

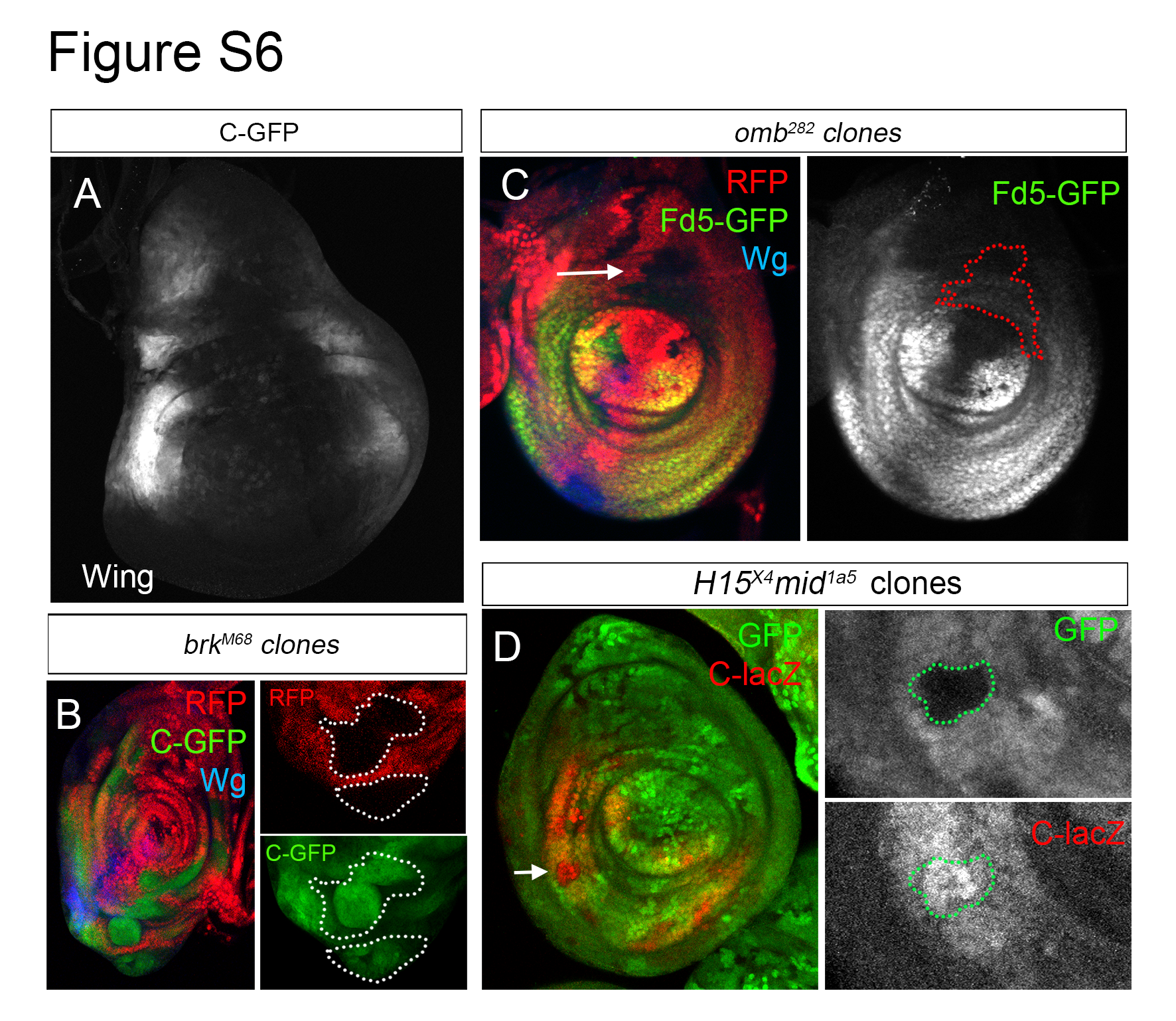

Supplement: Supplementary Figure 6 — brk, omb, and mid/H15 mutant clones have no effect on fd4/fd5 expression. (A) C-GFP activity is observed in the wing imaginal disc in a complementary pattern to dpp expression and similar to brk (not shown). (B) brkM68 mutant clones in the leg disc marked by the absence of RFP (red) and stained for C-GFP (green). Separate channels for RFP and C-GFP are shown and the brkM68 mutant clones are outlined by white dots. (C) Leg imaginal disc with a dorsal omb282 mutant clone marked by the absence of RFP (red, arrow) and stained for Fd5-GFP (green) and Wg (blue). Separate channel for Fd5-GFP is shown and the clone is outlined by a red dotted line. (D) H15X4 mid1a5 mutant clones marked by the absence of GFP (green) in a third instar leg imaginal disc stained for C-lacZ (red) and Wg (blue). An arrow marks a ventro-lateral anterior compartment clone and a close up view is shown. All clones were generated 48–72 h before dissection. [file Image_6.TIF]
